# The known and unknown of post-pump chorea: a case report on robust steroid responsiveness implicating occult neuroinflammation

**DOI:** 10.3389/fimmu.2024.1458022

**Published:** 2024-09-10

**Authors:** Muhammad Iqbal, Muizz Zaman, Niranjan Ojha, Yung-Tian A. Gau, Eufrosina I. Young

**Affiliations:** ^1^ Department of Neurology, SUNY Upstate Medical University, Syracuse, NY, United States; ^2^ Department of Neuroscience, Johns Hopkins School of Medicine, Baltimore, MD, United States

**Keywords:** chorea (non-Huntington’s), post-pump chorea, cardiopulmonary bypass (CPB), neuroinflammation, bioprosthetic aortic valve, hypoxia inducible factor

## Abstract

Post-pump chorea (PPC) is characterized by the development of choreiform movements following cardiopulmonary bypass (CPB) surgery. PPC occurs almost exclusively in children, and its pathophysiology remains unclear. Here we present an adult case of PPC after bovine aortic valve replacement (AVR) which exhibited dramatic and reproducible response to steroid, suggesting the presence of occult neuroinflammation. This observation suggests a novel underlying mechanism in certain subgroups of PPC, which is likely a heterogeneous condition to start with. Further research into the pathomechanisms of PPC could offer insights into managing this otherwise symptomatic control-only condition.

## Introduction

Choreiform movement, when seen after surgeries on cardiopulmonary bypass (CPB) pump, is known as post-pump chorea (PPC) (1, 2). Besides generalized chorea, comorbid encephalopathy and behavioral changes have been reported (1, 2). The first case series describing PPC was published in the 1960s, about children experiencing extrapyramidal symptoms and ultimately death after congenital heart surgeries with deep hypothermia and circulatory arrest (3). PPC is now well-characterized in the pediatric population, with an estimated incidence of 1.1–1.2% (2, 4). Conversely, PPC was not recognized in adults until the 2000s, as a rare complication with an incidence of 0.046% (2 out of 4,345 cases) in a single-center study (1, 5).

The clinical features and disease course of PPC are highly variable, with few consistent characteristics identified (1, 2, 4, 6). Abnormal movements, in a majority of cases generalized chorea, usually appear within 14 days post-surgery (3–12 days in children, 1–14 days in adults) after a latency period of 1–7 days (1, 2, 6). In children, additional symptoms such as orofacial dyskinesia, postural instability, dysphagia, and dysarthria can occur (2, 6). Common risk factors for PPC include deep hypothermia (below 25°C), rapid rewarming (above 0.18°C/minutes), long bypass time (over one hour), and extended total arrest (more than 45 min) (1, 2, 6). PPC lacks definite biomarkers and hence is a diagnosis by history and exclusion, except that at times (estimated around 38.8% in one study) magnetic resonance imaging (MRI) may reveal symmetrical caudate-putamen T2 hyperintensity (1, 2, 6). Regarding the disease course, approximately half of the cases resolve completely, while the remainder become persistent and irreversible (1, 2, 6). MRI changes may be associated with a higher likelihood of persistent diseases; poor outcome with neurocognitive developmental disorder can be seen in children, but age by itself is not an outcome predictor (1, 2, 6–12). Treatment primarily focuses on symptom control, traditionally with antipsychotics, anti-seizure medications, and benzodiazepines and more recently with vesicular monoamine transporter 2 (VMAT2) inhibitors (1, 2, 6). Out of the available VMAT2 inhibitors, tetrabenazine have demonstrated some efficacy, providing partial relief in two adult PPC cases (7, 11). For medically refractory individuals, deep brain stimulation targeting the globus pallidus internus may be considered (13, 14).

The underlying pathogenesis for PPC remains elusive (7). Hypoxia, microthromboembolism, metabolic insults, and acquired acanthocytosis have been proposed as potential mechanisms (15). However, these hypotheses do not fully explain the characteristics of PPC, such as the differences in children *vs.* in adult, not to mention that most cases lack etiology-specific pathognomonic findings (1, 7–12). Here, we present an adult male who developed PPC after an on-pump bovine aortic valve replacement (AVR). His clinical course suggests an inflammatory mechanism which may provide insight into some of the unknown aspects of PPC.

## Case description

**Figure 1:**
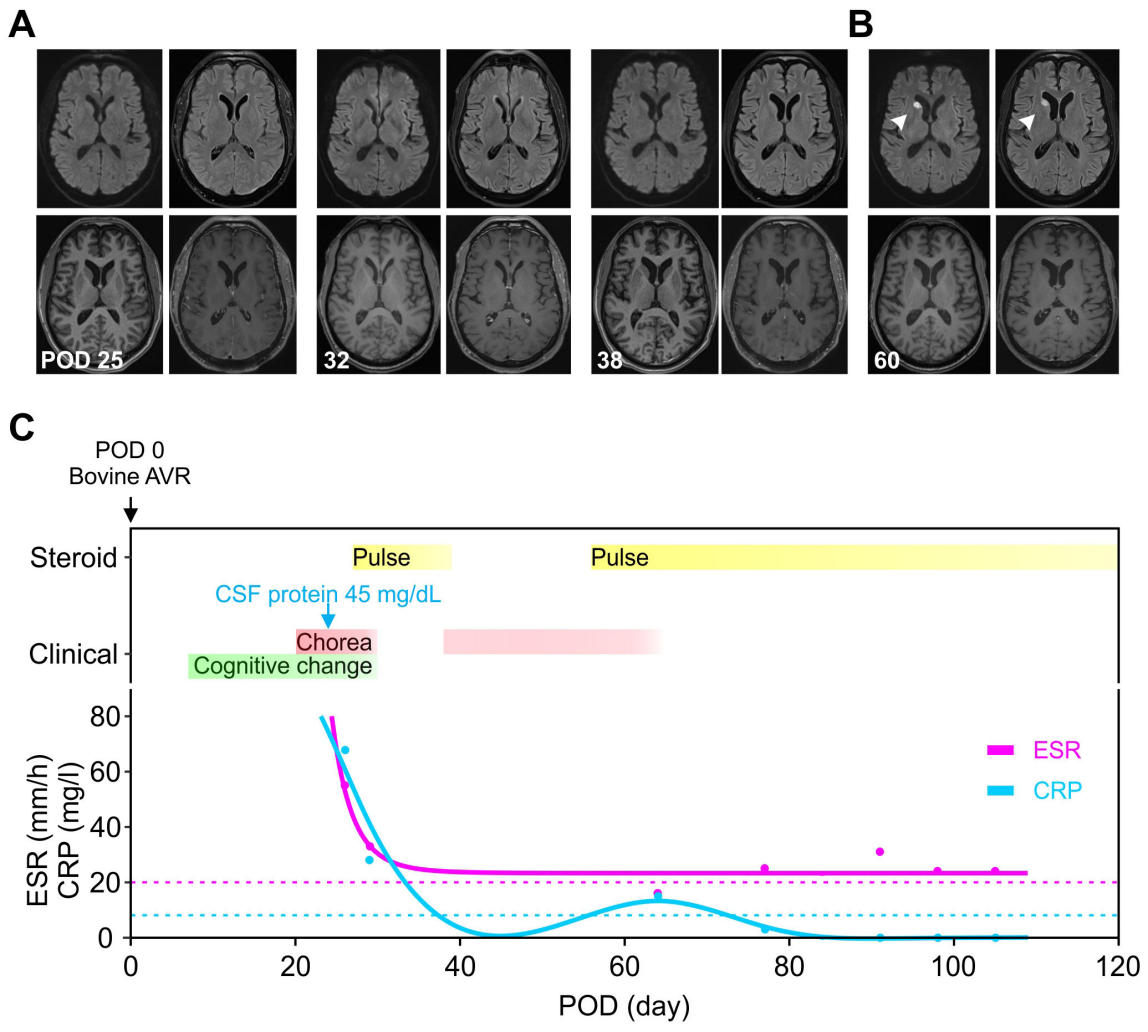
Evolution of clinical presentation, inflammatory biomarkers and imaging findings. **(A)** The MRIs were unremarkable on three occasions, including when the symptoms were most severe (POD 25) and during the recurrence (POD 38). **(B)** An acute ischemic stroke at the head of the right caudate (white arrowhead) was seen incidentally on POD 60 when the chorea was resolving. **(C)** The timing of immunomodulation (yellow) and clinical symptoms (red and green) was plotted against the days after valve replacement. The initial presentation began with cognitive changes (green, POD 7) and chorea (red, POD 14), then with symptom resolution on POD 30 during the pulse steroid (POD 27–31). Oral steroid was stopped on POD 39 with symptoms recurrence around POD 46 and then at maximum around POD 54–55. The second course of pulse steroid lasted from POD 56–60 with significant clinical improvement by POD 58. The inflammatory markers ESR (magenta) and CRP (cyan) were trended (dotted lines denote upper limit of normal range). AVR, aortic valve replacement; CRP, C-reactive protein; ESR, erythrocyte sedimentation rate; POD, postoperative day.

DD, a 66-year-old otherwise healthy gentleman, received a bovine AVR for aortic stenosis. Surgery was performed under mild hypothermia (30–32°C), with 72 minutes on-pump and 62 of total arrest. Out of the surgery he appeared encephalopathic, initially presumed to be perioperative stress and sedatives/analgesia. However, his mentation did not improve with time and instead progressed to the extent that, by postoperative day (POD) 7, his family was alarmed by the out-of-character hypervigilance and aggression (**Figure 1C**). By week three, he exhibited abnormal movement of frontal, bucco-lingual, axial, and appendicular chorea-ballismus, manifesting as flow of forehead wrinkling, eye closure, chewing/smacking, and jerking of extremities (**Supplementary Video 1**). These movements were involuntary, unpredictable, non-suppressible, non-distractible, and absent in sleep. Motor, sensory, and cerebellar functions were intact, specifically no oculomotor disturbance, loss of tone, dysarthria or dysphagia. Examination of other systems is unremarkable, including absence of constitutional symptoms or rheumatological stigmata. There was no movement disorder running in the family.

Serum hematological, metabolic, and infectious studies were normal, except for the elevated C-reactive protein (CRP) and erythrocyte sedimentation rate (ESR) (**Table 1**, **Figure 1C**). Survey for relevant toxins or environmental exposure also returned negative. Computed tomographic angiogram and MRI showed no vascular or structural lesions (**Figure 1A**, POD 25). Spot video-electroencephalography did not reveal epileptogenicity. A comprehensive panel checking other inflammatory and autoimmune markers was unremarkable (**Table 1**). Cell counts and glucose in the cerebrospinal fluid (CSF) were normal with protein at the upper limit (**Table 2**, **Figure 1C**). Advanced CSF tests for infectious, demyelinating, inflammatory, autoimmune, paraneoplastic, and degenerative markers did not yield any etiology (**Table 2**).

**Table 1:** Serological tests for common causes of chorea.

Test name	Result
Basic metabolic panel	
Sodium	134 mmol/L
Potassium	4.2 mmol/L
Bicarbonate	25 mmol/L
Chloride	98 mmol/L
Blood Urea Nitrogen	12 mg/dL
Creatinine	0.91 mg/dL
Glucose	94 mg/dL
Calcium, total	9.2 mg/dL
Magnesium	2.3 mg/dL
Hepatic function panel	
Alanine transaminase	35 u/L
Aspartate transaminase	44 u/L
Alkaline phosphatase	74 u/L
Albumin	4.1 g/dL
Bilirubin, total	0.6 mg/dL
Bilirubin, direct	<0.2 mg/dL
Complete blood count	
White blood cells	12.1*10^3^/µL
Platelets	377 *10^3^ /µL
Hemoglobin	14.2 g/dL
Hematocrit	42.2 %
Red blood cells	4.66 *10^6^/µL
Mean corpuscular volume	90.6 fL
Mean cell hemoglobin	30.4 pg
Mean corpuscular Hb concentration	33.6 g/dL
Red cell distribution width	14.5 %
Acanthocyte	None
International normalized ratio	1.1
Activated partial thromboplastin time	27 s
Hemoglobin a1c	5.9 %
Thyroid stimulating hormone Free thyroxine	2.82 U/mL 1.50 ng/dL
**Erythrocyte sedimentation rate**	**55 mm/hr (0–20)**
**C-reactive protein**	**67.8 mg/L (0–8)**
Copper serum	Normal
Ceruplasmin, serum	Normal
Vitamin B1	117.3 nmol/L
Vitamin B12	912 pg/ml
Vitamin B6	6.4 µg/L
Lyme IgG/M	Negative
Syphilis IgG/M	Nonreactive
AMPA-R antibody	Negative
Amphiphysin antibody	Negative
ANA titer and IFA	Negative
Anti-glial nuclear antibody type1	Negative
Anti-neuronal nuclear antibody type1	Negative
Anti-neuronal nuclear antibody type 2	Negative
Anti-neuronal nuclear antibody type 3	Negative
β2 glycoprotein IgG	<6.4 U/mL
β2 glycoprotein IgM	<1.1 U/mL
β2 glycoprotein IgA	5.6 U/mL
Cardiolipin IgA antibody	9.1 U/mL
Cardiolipin IgG antibody	<2.6 U/mL
Cardiolipin IgM antibody	1.4 U/mL
CASPR2 IgG	Negative
Centromere antibody	6 U/mL
CRMP5 IgG	Negative
Diluted Russell viper venom test	Negative
DPPX antibody IFA	Negative
dsDNA antibody	2 U/mL
GABA-B-R antibody	Negative
GAD65 antibody	0.00 nmol/L
GFAP antibody	Negative
Hexagonal phase phospholipid	Negative
Histone antibody	17 U/mL
IgLON5 antibody	Negative
JO-1 autoantibody	9 U/mL
LGl1-igG antibody	Negative
mGluR1 antibody	Negative
NIF antibody	Negative
NMDA-R antibody	Negative
Platelet neutralization procedure	Negative
Purkinje cell cytoplasmic antibody type 1	Negative
Purkinje cell cytoplasmic antibody type 2	Negative
Purkinje cell cytoplasmic antibody type TR	Negative
RNP autoantibody	11 U/mL
SCL-70 autoantibody	6 U/mL
Smith autoantibody	9 U/mL
SSA autoantibody	9 U/mL
SSB autoantibody	9 U/mL
Streptolysin O antibody	22 U/mL
Thyroid peroxidase antibody	0.3 U/mL

Significant findings are highlighted in bold.

**Table 2:** Cerebrospinal fluid workup.

Test name	Result
Differential count	
Red blood cell	<2 /uL
Total nucleated cell	<3 /uL
**Protein**	**45 mg/dL (15–45)**
Glucose	66 mg/dL
Appearance	Clear and colorless
AGNA-1	Negative
AMPA-R Ab CBA	Negative
Amphiphysin Ab	Negative
ANNA-1	Negative
ANNA-2	Negative
ANNA-3	Negative
CASPR2-IgG CBA	Negative
DPPX Ab IFA	Negative
GABA-B-R Ab CBA	Negative
GAD65 Ab Assay	Negative
GFAP IFA	Negative
IgLON5 IFA	Negative
LGI1-IgG CBA	Negative
mGluR1 Ab IFA	Negative
NIF IFA	Negative
NMDA-R Ab CBA	Negative
PCA-1	Negative
PCA-2	Negative
PCA-Tr	Negative
Escherichia coli K1	Negative
Hemophilus influenzae	Negative
Listeria monocytogenes	Negative
Neisseria meningitidis	Negative
Streptococcus agalactiae	Negative
Streptococcus pneumoniae	Negative
Cytomegalovirus	Negative
Enterovirus	Negative
Herpes simplex virus 1	Negative
Herpes simplex virus 2	Negative
Human herpesvirus 6	Negative
Human parechovirus	Negative
Varicella zoster virus	Negative
Cryptococcus neoformans/gattii	Negative

Significant findings are highlighted in bold.

The behavior and movement continued to worsen despite aggressive symptomatic control, and course was further complicated by total insomnia. His continuous video-electroencephalography demonstrated mild, generalized slowing without epileptogenicity, consistent with non-specific encephalopathy. We eventually initiated dexmedetomidine infusion for sedation and movement control. Forty-eight hours after, DD was able to resume prior sleep routine and gradually behavioral improvement, with nevertheless persistent and later nearly continuous choreiform movement unresponsive to clonazepam (0.5 mg twice daily [BID]) and valproic acid (1000–2250 mg) hitherto plus trials of quetiapine (25–100 mg for 13 days) and risperidone (0.25–2 mg for 12 days). It is worth noting that his behavioral changes rendered the use of VMAT2 inhibitors unfavorable.

In light of elevated inflammatory markers and high-normal CSF protein, we empirically started him on intravenous pulse methylprednisone (1000 mg) for five days from POD 27–31. This led to remarkable improvement with minimal symptoms at POD 30, associated with normalization of CRP and ESR (**Figure 1C**). Follow-up MRIs from POD 32 and 38 did not show any significant change (**Figure 1A**). Due to acute gastrointestinal bleed, he was unable to complete a 10-day short prednisone taper. Despite concomitant use of clonazepam 0.5 mg BID and valproic acid 500 mg BID, upon discontinuation of steroid, chorea recurred immediately within the same week (POD 46) along with the elevation of inflammatory markers (**Figure 1C**). A second round of pulse methylprednisone (POD 56–60) was administered with his abnormal movement visibly decreased in magnitude and frequency (POD 58) along with the course of steroid. This allowed symptom resolution and discharge to an acute rehabilitation facility upon completion of the pulse steroid. We had him on an extended prednisone taper with 10 mg decrements every 7 days, for a total of 6 weeks, along with the clonazepam 0.5 BID maintained for symptomatic control. The fourth MRI from POD 60 revealed incidentally an acute infarct at the right caudate (**Figure 1B**). He otherwise remained in full remission at his 3, 6, & 9-month outpatient follow-up (**Figures 1C**).

## Discussion

PPC, the emergence of chorea following CPB, was initially described in children and recently identified as a rarity in adults; to date, 18 cases of adult-onset PPC have been reported (1, 5, 7, 9–11, 15–19). Existing literature helps identify several consistent features (1, 5, 7, 9–11, 15–19). In concordance with these reports, our case developed early cognitive change and shortly after, generalized choreiform movement around POD 14 (1, 20). He experienced prolonged bypass duration and circulatory arrest, both known risk factors for PPC (1, 20). An extensive workup ruled out other etiologies (1, 2, 4). Therefore, we conclude that this presentation is consistent with an adult-onset PPC after bovine AVR, and that the distinct and robust response to one particular type of treatment, in contrast to the commonly-observed variable outcomes, may implicate an underlying singular pathomechanism.

The prevailing theory of PPC pathogenesis involves cerebral vasoconstriction and increased blood viscosity due to hypoxia, alkalosis, hypothermia, and/or rewarming, despite the lack of supportive evidence (1, 2, 4, 7). Our case demonstrated a reproducible immunosuppression response in symptoms and biomarkers (**Figure 1C**), suggesting neuroinflammation, either primary neural or secondary to systemic, could be a potential pathophysiology. As a matter of fact, neuroinflammation has been reported in various other post-CPB surgery neurological complications, such as chronic neurocognitive impairment or progressive supranuclear palsy-like Mokri syndrome (21–24).

It is well-recognized that cardiac surgery with bypass induces systemic and cerebral inflammation, leading to disrupted blood-brain-barrier and cellular injury (25). The inflammation may result from “contact activation” of maladaptive cascades in response to the internal surfaces of CPBs (26). Additionally, nonhuman extracellular matrix molecules such as α-1,3-galactose or N-glycolylneuraminic acid have been implicated in immune-related graft failure, potentially making the bovine bioprosthesis an immunogenic source (27, 28). Lastly, metabolic-immune signaling such as the hypoxia-inducible factor-1α pathway have also been shown to shift the immune balance toward a pro-inflammatory state (29, 30). Perioperative ischemia and reperfusion could induce such pathways, eventually contributing to the neural dysfunction observed in PPC.

PPC likely represents a heterogeneous condition, and the proposed inflammatory mechanism, along with other previously noted pathogenic processes, provides a more comprehensive understanding (1, 2, 4, 26–30). For example, deep hypothermia and circulatory arrest, both known risk factors for PPC, have been linked to heightened neuroinflammation, manifested by increased pro-inflammatory cytokines in the blood and CSF (31–33). The neuroinflammatory mechanism may also elucidate some of the distinctions between PPC in children and adults (1, 2, 4, 5). Children, having limited antigen exposure *a priori*, are believed to have less mature immune systems and more prone to autoimmunity and inflammation (34, 35). The proposed mechanism may also provide justification for the highly-variable course, particularly the late-onset and/or persistent cases, considering that the bovine tissue can continue being an antigen-presenting source long after surgery (1, 2, 4, 6, 27, 28). Furthermore, cerebral inflammation can lead to vascular injury or plasma hyperviscosity, both recognized risks for small vessel occlusion, potentially serving as a reciprocal feed-forward mechanism in the persistent PPC case (36, 37). The delayed right caudate infarct observed on POD 60 (**Figure 1A**), though incongruent in time course and unilaterality with the clinical presentation, may reflect the indolent nature of this process.

In summary, PPC is a heterogeneous collection of diseases with various mechanisms (38). The remarkable response to immunosuppression demonstrated in this case may thus suggest a subgroup of PPC with immune/inflammation-related chorea (1, 2, 6). Given the constraint of a single case and the lack of a control group, our preliminary observation requires cautious interpretation without presuming the causality. Further large-scale investigations into the pathophysiology and management of PPC are needed (39–41).

## Conclusions

We present a case of adult-onset PPC with remarkable and reproducible responsiveness to steroid treatment. This phenomenon suggests an underlying neuroinflammatory or neuroimmunological mechanism. We propose possible causes of inflammation and immunogenicity and illustrate how this hypothesis can address the unknown aspects of PPC. Further studies based on this observation may advance the understanding of its pathophysiology and targeted management.

## Data Availability

The original contributions presented in the study are included in the article/**Supplementary Material**. Further inquiries can be directed to the corresponding authors.

## Glossary

AVR: Aortic valve replacement
BID: twice daily
CPB: Cardiopulmonary bypass
CSF: Cerebrospinal fluid
MRI: Magnetic resonance imaging
POD: Postoperative day
PPC: Post-pump chorea
